# Correction: İlhan et al. Exploratory Analysis of Circulating Serum miR-197-3p, miR-1236, and miR-1271 Expression in Early Breast Cancer. *Int. J. Mol. Sci.* 2025, *26*, 8944

**DOI:** 10.3390/ijms27094138

**Published:** 2026-05-06

**Authors:** Burak İlhan, Sibel Kuraş, Berkay Kılıç, Ceren Tilgen Yasasever, Hilal Oğuz Soydinç, Hani Alsaadoni, Gözde Öztan, Arash Adamnejad Ghafour, Muhammed Ucuncu, Enver Kunduz, Süleyman Bademler

**Affiliations:** 1 Department of Surgery, Istanbul Faculty of Medicine, Istanbul University, 34093 Istanbul, Türkiye; burak.ilhan@istanbul.edu.tr; 2 Department of Medical Biochemistry, Hamidiye Faculty of Medicine, University of Health Sciences, 34668 Istanbul, Türkiye; sibel.kuras@sbu.edu.tr; 3 Department of Surgery, Oncology Institute, Istanbul University, 34093 Istanbul, Türkiye; berkaykilic28@yahoo.com; 4 Department of Basic Oncology, Oncology Institute, Istanbul University, 34093 Istanbul, Türkiye; ceren.yasasever@istanbul.edu.tr (C.T.Y.); hoguz@istanbul.edu.tr (H.O.S.); 5 Department of Medical Biology, International School of Medicine, University of Health Sciences, 34668 Istanbul, Türkiye; hani.alsaadoni@sbu.edu.tr; 6 Department of Medical Biology, Istanbul Faculty of Medicine, Istanbul University, 34093 Istanbul, Türkiye; gozdeoztan@istanbul.edu.tr; 7 Department of Cancer Genetics, Oncology Institute, Istanbul University, 34093 Istanbul, Türkiye; arash.adamnezhad@gmail.com; 8 Department of Anesthesia, Vocational School of Health Services, Istanbul Gelisim University, 34310 Istanbul, Türkiye; muhammeducuncu@gmail.com; 9 Department of Surgery, Faculty of Medicine, Bezmialem Foundation University, 34093 Istanbul, Türkiye; drkunduz@yahoo.com

The following reference [45] has been retracted and should be replaced from the original publication [1]:

45. Huang, S.; Huang, P.; Wu, H.; Wang, S.; Liu, G. LINC02381 aggravates breast cancer through the miR-1271-5p/FN1 axis to activate PI3K/AKT pathway. *Mol. Carcinog.* **2022**, *61*, 346–358.

In order to maintain accuracy and precision, a correction has been made to the References section, where Reference [45] has been updated as follows:

45. Perez-Moreno, E.; Ortega-Hernandez, V.; Zavala, V.A.; Gamboa, J.; Fernandez, W.; Carvallo, P. Suppression of breast cancer metastatic behavior by microRNAs targeting EMT transcription factors. A relevant participation of miR-196a-5p and miR-22-3p in ZEB1 expression. *Breast Cancer Res. Treat.* **2025**, *212*, 277–290.

Consequently, the sentence containing the original references [45] in Section 3, Discussion, Paragraph Number 5, has also been updated:

In the literature, a recent study suggests that miR-1271 inhibits cell invasion and proliferation in breast cancer cells by negatively regulating transcription factors such as ZEB1 and SLUG/SNAI2 (Snail family transcriptional repressor 2 gene) of EMT [45]. Although previously implicated in mTOR signaling and treatment resistance for various cancers, miR-1271 did not show diagnostic relevance in our cohort, potentially due to tumor subtype, heterogeneity, or its role in later disease stages.

The authors state that the scientific conclusions are unaffected. This correction was approved by the Academic Editor. The original publication has also been updated.
